# Joint Pacing and Vascular Intervention for the Management of Cardiac Device Associated Central Venous Obstruction

**DOI:** 10.1111/pace.70019

**Published:** 2025-07-31

**Authors:** Nadeev Wijesuriya, Helen Sinabulya, Helena Johann‐Meyer, Keisha Kellman, Felicity de Vere, Sandra Howell, Alphonsus Liew, Paolo Bosco, Steven A. Niederer, Stephen Black, Christopher A. Rinaldi

**Affiliations:** ^1^ King's College London London UK; ^2^ Guy's and St Thomas's NHS Foundation Trust London UK; ^3^ Karolinska Institutet Stockholm Sweden; ^4^ Imperial College London UK; ^5^ Alan Turing Institute London UK

**Keywords:** extraction, pacemaker, superior vena cava syndrome, venoplasty, venous obstruction, venous stenting

## Abstract

**Background and Aims:**

Central venous obstruction (CVO) increases the complexity of pacing interventions, whether it be with device‐associated symptomatic superior vena cava syndrome (SVCS), or by impeding new implants. Endovascular treatment involves the joint expertise of both cardiac pacing and vascular specialists. We report the outcomes of such procedures at our institution.

**Methods:**

A single‐center retrospective observational study, examining outcomes of joint pacing‐vascular procedures for CVO. Cases were screened from an existing institutional database.

**Results:**

There were 19 total cases. Two were new device implants where the novel “inside‐out” procedure was utilized to establish access in SVCS, both with no complications. The remainder (*n* = 17) were transvenous lead extractions plus attempted recanalization of CVO using venoplasty with or without stenting. Transvenous devices were re‐implanted in eight patients. Complete procedure success rate was 84%. There were two cases of pericardial effusion requiring pericardiocentesis, resulting in procedure abandonment. There was no in‐hospital mortality and no cases of emergency sternotomy. Over mean follow‐up of 28 months, 2/6 patients receiving venoplasty (33%) and 2/8 patients receiving stenting (25%) required re‐intervention for symptomatic restenosis. Of the patients who were not re‐implanted with a transvenous device following initially successful endovascular intervention (6/14), none had recurrence over the follow‐up period.

**Conclusion:**

Pacing interventions in SVCS carry a significant risk profile, requiring management by experienced operators in high‐volume centers to maximize safety. Endovascular interventions have a significant recurrence rate, with up‐front stenting potentially being superior. Our data suggests that those without re‐implantation of transvenous leads may have better long term outcomes.

**Abbreviations**
CIED: cardiac implantable electronic device
CRT: cardiac resynchronization therapy
CVO: central venous obstruction
SVCS: superior vena cava syndrome
TLE: transvenous lead extraction

## 1 Introduction

Venous occlusion or stenosis of the great chest veins is a common consequence of transvenous pacing, with reported incidences of up to 30% [1]. It is postulated that repetitive chronic friction between the pacing lead and endothelium initiates an inflammatory process resulting in fibrosis and/or thrombosis [2]. Additionally, the subsequent development of collateral circulation may cause further slowing in the affected vein, leading to a prothrombotic state [3]. The rate of symptomatic venous occlusion has been reported as roughly 6% in device recipients [4], with the incidence of the most serious venous complication, superior vena cava obstruction (SVCS), ranging from 1/3100 to 1/650 [3]. A major predictive factor for development of venous stenosis is increasing number of transvenous leads [5], a phenomenon which is likely to become more prevalent as pacing guidelines have expanded the use of cardiac resynchronization therapy (CRT) to incorporate larger cohorts of patients, such as those with mild/moderate left ventricular (LV) dysfunction [6] or those receiving atrioventricular (AV) node ablation [7].

The management of pacing‐associated venous occlusion has evolved over the years. While initially systemic anticoagulation was the treatment of choice, interventional options have become more common due to the emergence of endovascular options for venoplasty and stenting [8], and the improved safety profile of transvenous lead extraction (TLE) [9]. TLE has a Class 1 recommendation from the Heart Rhythm Society (HRS) consensus statement for the management of symptomatic SVCS [10]. That being said, there is a paucity of large data evaluating the management of such patients in the setting of pacing intervention, primarily limited to case reports and small cohorts [11, 12, 13]. We aimed to add to the body of evidence in this important field by reporting on our institution's experience of joint cardiac and vascular intervention in the management of venous occlusion in pacing procedures.

## 2 Methods

### 2.1 Patient Selection

Research was conducted in accordance with the Declaration of Helsinki, with institutional review board approval. Our existing single‐center retrospective registry of TLE from 2008 to 2025 was initially screened for patients coded with “vein stenosis” or “vein occlusion.” These patients’ electronic health records were manually screened for those having received a joint pacing and vascular intervention for the treatment of venous occlusion. For identified patients, baseline characteristics, procedural data and outcomes were extracted from the existing registry and electronic health records.

### 2.2 General Procedure

All extraction and joint surgical cases in our center are discussed at a multidisciplinary meeting prior to the procedure, with input from cardiologists, cardiothoracic, and vascular surgeons. All cases undergo pre‐procedure CT venography. Joint cases are performed in a dedicated hybrid theatre, with cardiothoracic cover and cardiac bypass on standby. Cases are performed under general anesthesia with transoesophageal echocardiographic (TOE) guidance and invasive hemodynamic monitoring.

### 2.3 Procedure‐Specific Methods—Venoplasty With or Without Stenting

**FIGURE 1:**
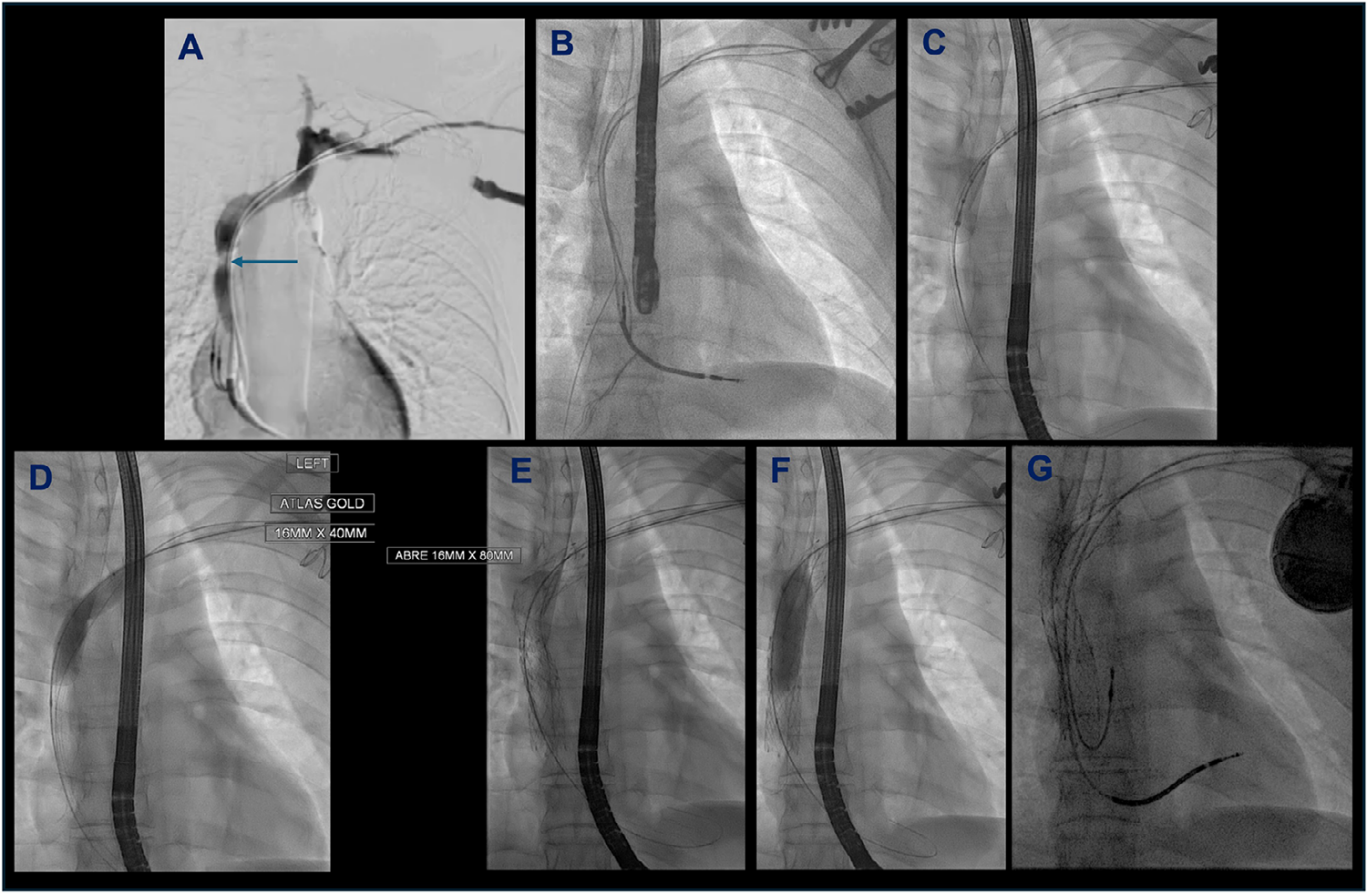
Workflow of case for extraction, venous stenting, and re‐implantation. (A) Digital subtraction angiography using contrast injection from left arm demonstrating SVC occlusion (arrow). Note also partial azygous filling; (B) baseline fluoroscopy; (C) following lead extraction, intravascular ultrasound performed to characterize lesion; (D) initial balloon venoplasty; (E) stent deployment; (F) stent post‐dilatation; (G) final image with transvenous leads re‐implanted. [Colour figure can be viewed at wileyonlinelibrary.com]

SVC venoplasty involved balloon angioplasty to dilate the stenotic or occluded segments. In these cases, guide wire access was established following TLE to cross the lesion. Intravascular ultrasound (IVUS) using the Philips Visions PV.035 digital IVUS catheter with or without contrast injection was used to delineate the lesion. Venoplasty was performed using a high‐pressure balloon catheter (Atlas Gold PTA Dilatation Catheter) that was positioned at the stenosis, inflated to the rated burst pressure, and then deflated. Stent (self‐expanding dedicated venous stent) deployment followed if recanalization was incomplete or restenosis was anticipated, as per operator discretion. The stent was then dilated with a similar high pressure balloon as used pre‐stenting. Following endovascular therapy, depending on indication, patients were either implanted with a replacement transvenous device, an upgraded transvenous device, a leadless device, or in some cases, not re‐implanted (Figure 1).

### 2.4 Procedure‐Specific Methods, Jugular‐Venous Bypass

In cases where endovascular treatment was unsuccessful a right atrium (RA) to the internal jugular vein (IJV) bypass was subsequently performed by vascular and cardiothoracic surgeons. This comprised a median sternotomy and neck incision exposing the IJV. The bypass was created using an appropriately sized polytetrafluoroethylene (PTFE) graft that was tunneled as deep in the mediastinum as possible, to avoid compression, and was anastomosed with end to side technique. Standard hemostatic techniques were performed. Post‐operative CT venography is used to confirm graft patency.

### 2.5 Procedure‐Specific Methods—“Inside‐Out” Procedure (IOP)

This novel procedure is used to establish thoracic venous access for new device implants in patients with SVCS. The concept has previously been described in detail [14, 15]. In short, the IOP reverses traditional techniques by directing a needle outward from within the body. From a femoral venous approach, a needle guide and wire (Surfacer Venous Access Tool, Merit Medical, South Jordan, UT) is advanced through the occlusion. At the level of the subclavian vein, an anterior puncture directed out avoids compromising any critical structures such as the lung or arteries. Once the wire is externalized, this allows standard peelable pacing sheaths to be advanced through the occlusion to establish superior transvenous access for pacing.

### 2.6 Statistical Analysis

Baseline characteristics are summarized by mean ± standard deviation (SD) for continuous variables with a normal distribution, median for variables with a skewed distribution, and frequency distribution for categorical variables. Analyses were conducted on Stata Statistical Software Package Release 18 (StataCorp LLC, College Station, TX).

## 3 Results

### 3.1 Patient Population

From our center's extraction registry (*n* = 1316), 34 patients were coded as having venous occlusion. Manual screening of these patients excluded 15 cases where TLE was utilized in patients with asymptomatic CIED‐associated subclavian obstruction to facilitate a device upgrade. We thus identified a final study cohort of 19 patients who received joint pacing‐endovascular interventions for symptomatic obstruction. The baseline characteristics are displayed in Table 1. The cohort was comprised of 58% males, with a mean age of 64 years. Nine patients had existing standard pacemakers, and 8 (42%) had a complex device (ICD/CRT‐P/CRT‐D). Three patients were pacing dependent (16%). The mean LV ejection fraction (LVEF) was 48%, and 42% of patients had baseline symptoms of heart failure. Fifteen patients (79%) had baseline features of venous obstruction: facial swelling (58%), upper limb swelling (53%), or shortness of breath (47%).

**TABLE 1:** Baseline characteristics.

Demographic variable	Study cohort (*n* = 17)
Age (years)	64.1 ± 15
Sex	
Male	11 (58%)
Female	8 (42%)
Existing device	
None	2 (11%)
Pacemaker	9 (47%)
ICD	4 (21%)
CRTP	1 (5%)
CRTD	3 (16%)
Pacing dependent	3 (16%)
Body mass index (kg/m^2^)	26.9 ± 5
Chronic kidney disease	3 (16%)
Diabetes	1 (5%)
Smoker	5 (26%)
Thrombophilia	1 (5%)
Atrial fibrillation	6 (32%)
Ischaemic heart disease	5 (26%)
Heart failure	8 (42%)
Left ventricular ejection fraction (%)	47.5 ± 13
Venous obstruction symptoms	Total 15 (79%)
Facial swelling	11 (58%)
Upper limb swelling	10 (53%)
Shortness of breath	9 (47%)

Abbreviations: CRTD, cardiac resynchronization therapy with a defibrillator; CRTP, cardiac resynchronization therapy pacemaker; ICD, implantable cardioverter‐defibrillator.

### 3.2 Procedure Characteristics

Detailed procedure characteristics are displayed in Table 2. The procedure indication was symptomatic device‐related SVCS in 14 patients. Two patients without existing devices had asymptomatic SVCS, and underwent the inside‐out procedure to establish superior venous access for new device implantation.

**TABLE 2:** Procedure characteristics.

Procedure characteristic	Study cohort (*n* = 17)
Indication	
New implant	2 (11%)
Symptomatic SVCS	14 (73%)
Subclavian vein occlusion	2 (11%)
Thoracic outlet syndrome	1 (5%)
Pacing intervention	
Extraction plus re‐implant	14 (73%)
Extraction, no re‐implant	3 (16%)
New implant	2 (11%)
Number of leads removed	37
Mean lead dwell time (years)	10.2±7.5
Endovascular intervention	
Planned but not performed	3 (16%)
Venoplasty	6 (31%)
Venoplasty plus stent	8 (42%)
Inside‐out puncture	2 (11%)
Extraction method (*n* = 17)	
Manual traction only	1 (6%)
Laser sheath	16 (94%)
Femoral approach	6 (35%)
Device upgraded (*n* = 17)	3 (18%)
New device implanted	
None	3 (16%)
Pacemaker	1 (5%)
ICD	2 (11%)
CRTP	2 (11%)
CRTD	5 (26%)
Conduction system pacing	1 (5%)
Leadless pacemaker	5 (26%)
Procedure duration (minutes, median)	177

Abbreviations: CRTD, cardiac resynchronization therapy with a defibrillator; CRTP, cardiac resynchronization therapy pacemaker; ICD, implantable cardioverter‐defibrillator; SVCS, superior vena cava syndrome.

Seventeen patients underwent TLE. Thirty‐five leads were removed (five redundant) with a mean dwell time of 10.2 years. TLE was laser‐assisted in the majority of cases (94%), with a femoral approach required in six patients (35%). To restore venous patency, venoplasty alone was used in 6 (35%) cases, with venous stenting utilized in 8 (47%).

Following endovascular intervention, devices were implanted/re‐implanted in 14/19 patients (74%). Three patients were implanted with upgraded devices, and five patients had leadless pacemakers placed.

Three patients did not have devices replaced. Two had dual chamber pacemakers originally implanted for sick sinus syndrome with a low pacing burden. One patient had a primary prevention ICD for non‐ischemic cardiomyopathy, with no detected arrhythmias following initial implant, and LVEF that had improved to 45% with medical therapy. For these three cases, a conservative approach following TLE was decided upon after appropriate counselling with the patients regarding their wishes, and heart team MDT discussion.

### 3.3 Acute Outcomes

Sixteen patients had complete procedural success (84%), with complete device management and venous patency restoration. Both inside‐out procedures were undertaken with no acute complications, and no complications on follow‐up.

Of the remaining 17 cases where joint TLE and endovascular venous intervention was planned, there was clinical extraction success in all patients. There were two pericardial effusions, both requiring emergent pericardiocentesis (11%).

In one case, effusion developed immediately following removal of 34‐year‐old pacing leads. Here, endovascular intervention was not attempted following drainage of the effusion, with the decision made to treat the stenosis at a later date. In the second case, TLE was successful, however effusion developed during attempted stenting, which was ultimately unsuccessful. In both of these cases, a leadless device was implanted rather that a transvenous one.

There was one post‐operative hematoma requiring evacuation, and one atrial lead displacement requiring in‐hospital revision. Two patients had intra‐operative tachyarrhythmia requiring pharmacological cardioversion. There were no cases of in‐hospital mortality, and no emergent sternotomies were required. The median inpatient hospital stay was 5 days.

### 3.4 Long‐Term Outcomes

**FIGURE 2:**
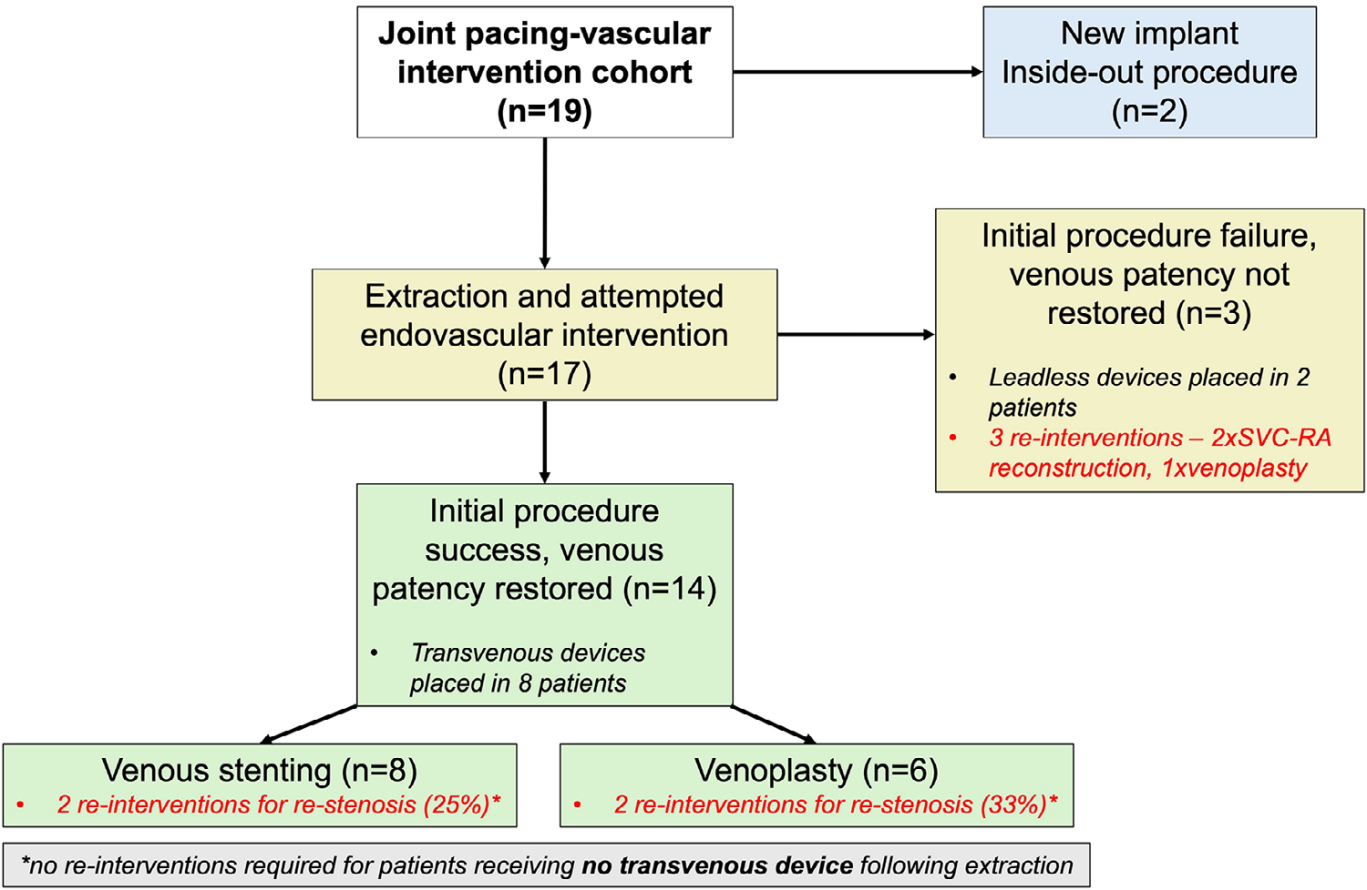
Outcomes of joint pacing‐vascular interventions. [Colour figure can be viewed at wileyonlinelibrary.com]

Long‐term outcomes are diagrammatically represented in Figure 2. The mean follow‐up duration was 28 months. Overall, seven patient's required subsequent intervention for venous stenosis. This included the three patients who did not undergo the planned endovascular intervention at initial procedure—two underwent subsequent SVC‐RA bypass procedures, with the other receiving a venoplasty.

Of the 14 patients who underwent initial TLE and endovascular intervention, a total of four patients required re‐intervention for symptomatic re‐stenosis (29%). 2/6 patients whose index procedure was venoplasty required re‐do venoplasty. 2/8 patients whose index procedure was stenting received subsequent venoplasty—one for in‐stent re‐stenosis, and one for stent outflow stenosis.

All patients requiring re‐do venoplasty procedures were those who had transvenous devices re‐implanted at initial procedure. The overall rate of re‐stenosis for patients receiving successful index TLE, endovascular intervention, and transvenous device re‐implantation was 4/8 (50%). In the case of venoplasty, the incidence of restenosis re‐intervention in this sub‐cohort was 2/3 (66%), and 2/5 in the case of stenting (40%).

There were no cases of mortality in the first 6 months following the index procedure. Over a mean follow‐up duration of 31 months, there was no procedure‐related mortality. There was one incidence of two major complications (acute lacunar stroke and phrenic nerve paralysis in the same patient) during an SVC‐RA bypass procedure.

## 4 Discussion

We present our experience of joint pacing and vascular interventions for the treatment of venous stenosis, both in the context of TLE and new implants. The majority of study patients had device‐associated SVCS. To our knowledge our experience represents the largest single‐center cohort reported to date. Our findings were as follows:

1. The rate of short‐term complete procedural success was 84%.
2. Acute procedural complications of pericardial effusion requiring pericardiocentesis occurred in 2/19 patients. There were no cases of procedure‐related mortality or emergency sternotomy.
3. There was a significant recurrence rate of symptomatic venous stenosis. Outcomes seem to be improved when (a) up‐front stenting was utilized rather than venoplasty alone and (b) when no transvenous leads were replaced crossing the stenosed segment.

### 4.1 Short Term Outcomes—Procedural Safety

Our data aligns with prior small cohort studies and pooled analyses which suggest a modest acute complication rate. In a pooled analysis of 108 patients from 74 studies examining SVCS after device implantation, the complication rate for endovascular interventions was 6.3% [16]. A recent cohort study of device‐associated SVCS published by Mekary et al. [17] reported a case of SVC tear and tamponade in a series of 15 patients. While robust conclusions are difficult to draw from smaller studies, TLE in the context of SVCS may have a higher risk profile than standard TLE procedures, which generally have a major complication rate of around <2% [9]. Indeed, in a cohort of 71 patients at our institution who received laser‐assisted TLE to facilitate upgrade in the context of CIED‐associated asymptomatic *subclavian* stenosis, the major complication rate was 3% [18].

Despite a higher risk, there were no instances of procedure‐related mortality or conversion to open surgery in our cohort. In these cases, it is vital that there is comprehensive MDT peer‐review pre‐procedure, as well as good communication with the patient regarding risk to enable informed shared decision making, including a detailed discussion of the cumulative risks for intervening on complications such as tamponade, and potential increased duration of hospital stay and recovery. It should be considered and communicated that for complex cases, such as those with severe SVO or multiple prior surgeries, surgical risk may be higher. Procedures should be performed in high volume centers by experienced operators, ideally in a hybrid theatre. Invasive hemodynamic monitoring and peri‐procedural TOE improve safety through detection of complications such as tamponade early, thereby enabling expedited intervention to avoid permanent morbidity.

### 4.2 Medium Term Outcomes—Efficacy of Endovascular Therapy: Venoplasty Versus Stenting

We report an overall recurrence rate of 25% for venous stenting, and 50% for venoplasty alone. This generally supports the data from prior studies. Riley et al. reported a 25% rate of recurrence following stenting at 5 year follow‐up in a pooled cohort analysis [16]. Similarly, Mekary et al. reported a recurrence rate of 17% following stenting [17]. For the most part, higher recurrence rates have been observed with venoplasty alone compared to stenting. Peters et al. reported a recurrence rate of 33% for venoplasty in a cohort of 18 patients with device associated venous stenosis, primarily brachiocephalic or axillary [19]. In a meta‐analysis of central venous obstruction in those with indwelling hemodialysis lines (*n* = 655), venoplasty had a pooled 12‐month primary patency rate of 38%, compared to 54% when a stent was deployed [20]. Of note, in this study, the primary patency rate was <30% at 5 years follow up with both venoplasty alone or with stenting. These findings align with those reported by Gur et al. [21], who reported that in an observational study of hemodialysis patients with symptomatic central venous obstruction (CVO), primary patency rates at 12 months were 59% with stenting compared to 42% with venoplasty. In this cohort, patency also declined over time to below 30% in both groups at 5 years.

It is perhaps unsurprising that stenting has more favorable outcomes than venoplasty. Intrinsic radial pressure is characteristic of endovascular stents, that prevents elastic recoil of the vessel, thus theoretically reducing the need for re‐intervention [20]. Why then, do patency rates appear not to hold in the long term? There may be several explanations for this. First, endovascular stents are generally designed for arterial rather than slow venous blood flow, and this may contribute to late failure. Perhaps more important is the role of collider bias in underestimating the efficacy of stenting compared to venoplasty. All current evidence in this field is observational—a specific flaw with the data thus far is that stenting has historically been reserved for more complex lesions, or used as an immediate salvage therapy those where venoplasty was not effective. It may be that if up‐front stenting was used for all lesions rather than just complex ones, we may see improved patency rates with this therapy. We note here that while stents have traditionally been reserved for difficult cases due to a perceived higher complication rate [22], recent pooled analyses have suggested a similar safety profile to venoplasty [20].

There are additional technical considerations that may influence the decision to stent or not. Principally in removing the leads with laser it is apparent that the venous stenosis that develops if no leads are replaced is significantly more difficult to cross than at the primary procedure. In the two patients that required open surgical bypass multiple attempts at crossing failed. The authors postulate that the effect of the laser leads to a much more aggressive fibrotic response with complete destruction of residual lumen if stenting is not undertaken. This may explain why higher rates of restenosis were observed with venoplasty alone our cohort, where laser was used for TLE in all but one patient. Further work to understand this is required.

We believe therefore, that our study adds to the growing evidence base that venous stenting in combination with TLE is a reasonable choice as a first‐line treatment option for CIED‐associated SVCS. However, it is important to keep in mind that using current technology, patency rates will decline over time. Indeed, the primary patency rates of 75% with stenting over the relatively short follow‐up duration of 28 months observed in our study are modest, highlighting the limitations of current technology. Further development of dedicated venous stents would be a valuable step in progressing this therapy. This is a vital consideration in this condition compared to malignant or dialysis‐associated SVCS, where life expectancies tend to be shorter. To provide holistic treatment, endovascular therapies must be combined with appropriate device management to maximized long‐term outcomes.

### 4.3 Maximizing Long Term Outcomes—Device Management in Venous Obstruction

While overall recurrence rates for endovascular therapy in the context of CIED‐associated obstruction are significant, most evidence is in agreement that outcomes are improved if no transvenous device is replaced across the stenosis. In our cohort, there were no observed recurrences in patients where the transvenous device was not replaced during the follow‐up period. Peters et al. reported that in their cohort, of the 10 patients with no recurrence following a single venoplasty procedure, eight had not been re‐implanted with a transvenous device [19]. Similarly, both patients in the Mekary et al. study [17] who were not re‐implanted experienced no symptom recurrence. These small numbers are supported by larger studies and meta‐analyses which show that incidence of venous obstruction is significantly associated with the number of transvenous leads and abandoned leads [5, 23].

As part of the decision‐making process, it is vital that the pacing and/or defibrillator indications are re‐visited at time of extraction. Those with sick sinus syndrome may elect to forgo device replacement if their symptoms have resolved and have minimal pacing burden, as was the case for two of our patients. In the era post‐DANISH trial [24], with appropriate counseling, those never having received therapy from a primary prevention ICD may potentially not be re‐implanted, especially if the LV has positively remodeled with contemporary medical therapies [25]. Similarly, another concept to reduce lead burden would be to consider extraction of redundant leads in the event of failure and necessity to implant a replacement. In general, physicians have adopted to add leads rather than extract and replace, as extraction is a more complex procedure with a higher risk profile, not available in all centers. With contemporary tools, acute complication rates are relatively low [26], and the risk‐benefit profile may now be tilted in favor of extracting all redundant leads at time of revision to reduce the risk of long‐term complications such as SVCS. Randomized trials in this space would be helpful in informing future practice. In addition, novel tools such as machine learning may help to inform individualized treatment plans by using imaging as simple as a plain chest radiography to predict risk of major complications during extraction [27].

Leadless pacing and defibrillator therapy have become a very attractive option for patients with CVO in recent years. Right ventricular leadless pacing with devices such as MICRA (Medtronic, Minneapolis, MN) and AVEIR (Abbott Laboratories, Chicago, IL) have a strong track record of reliability [28], with atrial leadless devices emerging to provide true dual chamber pacing [29]. Subcutaneous ICD's are a reliable option for patients who pass screening, with the most contemporary iteration, the Medtronic extravascular ICD, able to provide defibrillator as well as anti‐tachycardia pacing therapy [30]. The WiSE‐CRT device (EBR Systems Ltd, Sunnyvale CA) provides a leadless option for CRT or conduction system pacing by providing ultrasound‐based leadless LV endocardial stimulation [31, 32]. There have been case reports of leadless devices being combined to provide a totally leadless CRT‐D [33].

While novel leadless technologies show promise and push the boundaries of what is feasible, more work is needed before widespread use can be adopted. Issues with device‐device interaction and how to manage implants at the time of generator replacement are as yet to be addressed. In addition, while the individual major complication rates of leadless implants are relatively low, the cumulative complication rate as multiple devices are added for functionality will be more significant. This needs to be taken into consideration during the shared decision‐making process, especially as novel transvenous treatments such as the inside‐out procedure are feasible options when performed in the right setting.

### 4.4 Limitations

This was a retrospective single center observational study. There are potential biases inherent to a retrospective cohort design. Selection bias is a possibility, given that patients were recruited from a single center with its own institutional criteria for intervention. This is a niche and highly heterogeneous patient group. As such, patient selection is on a case‐by‐case basis rather than a process which is more protocolized. This may limit the generalizability of the findings. Potential misclassification of variables or outcomes is also a possibility, as this study utilized data from existing medical records. Furthermore, there is potential for confounding variables affecting the strength and direction of associations in a small cohort such as this. In addition, the follow‐up duration of 28 months limits the conclusions that can be drawn about the efficacy of these interventions over the long‐term.

The small sample size also limits the strength of the conclusions that can be drawn, especially as statistical analysis is restricted in cohorts of this size.

Nevertheless, this study adds to a body of evidence in this field which is predominantly comprised of case reports and smaller case series. SVCS is relatively uncommon, and encompasses a very diverse patient population. Due to the broad variety of patient characteristics and need for long‐term outcomes, randomized control trials in this space would be difficult to conduct. An international prospective registry would be useful in providing more robust outcome data, which would mitigate the potential bias and limitations of a retrospective study design.

## 5 Conclusion

Our study supports the use of extraction combined with up‐front venous stenting for the treatment of symptomatic SVCS, provided this is performed by experienced high‐volume operators. This is not a benign procedure, and careful pre‐procedural planning, communication between cardiac and vascular teams, and thorough patient counseling are vital. Long‐term patency rates with current technology are modest, and appropriate device management to reduce transvenous lead burden is imperative for holistic treatment of this complex patient population.

## Conflicts of Interest

The authors declare no conflicts of interest.

## Data Availability

The data that support the findings of this study are available from the corresponding author upon reasonable request.

## References

[1] G. M. Calvagna and S. Patanè , “Pacing Venous Occlusion,” International Journal of Cardiology 181 (2015): 42–45, 10.1016/J.IJCARD.2014.11.188/ASSET/61592718-F9CA-4A30-A2AE-4D4B50B55ECE/MAIN.ASSETS/GR1.SML. 25481312

[2] A. Kutarski , R. Pietura , K. Młynarczyk , B. Małecka , and A. Głowniak , “Pacemaker Lead Extraction and Recapture of Venous Access: Technical Problems Arising From Extensive Venous Obstruction,” Cardiology Journal 19, no. 5 (2012): 513–517, 10.5603/CJ.2012.0093. 23042316

[3] G. Rozmus , J. P. Daubert , D. T. Huang , S. Rosero , B. Hall , and C. Francis , “Venous Thrombosis and Stenosis After Implantation of Pacemakers and Defibrillators,” Journal of Interventional Cardiac Electrophysiology 13, no. 1 (2005): 9–19, 10.1007/S10840-005-1140-1/METRICS. 15976973

[4] S. S. Do Carmo Da Costa , A. Scalabrini Neto , R. Costa , J. Guilherme Caldas , and M. Martinelli Filho , “Incidence and Risk Factors of Upper Extremity Deep Vein Lesions After Permanent Transvenous Pacemaker Implant: A 6‐Month Follow‐Up Prospective Study,” Pacing and Clinical Electrophysiology 25, no. 9 (2002): 1301–1306, 10.1046/J.1460-9592.2002.01301.X. 12380764

[5] C. Suga , D. L. Hayes , L. K. Hyberger , and M. A. Lloyd , “Is There an Adverse Outcome From Abandoned Pacing Leads?,” Journal of Interventional Cardiac Electrophysiology 4, no. 3 (2000): 493–499, 10.1023/A:1009860514724. 11046188

[6] A. B. Curtis , S. J. Worley , P. B. Adamson , et al., “Biventricular Pacing for Atrioventricular Block and Systolic Dysfunction,” New England Journal of Medicine 368, no. 17 (2013): 1585–1593, 10.1056/NEJMoa1210356. 23614585

[7] M. Brignole , F. Pentimalli , P. Palmisano , et al., “AV Junction Ablation and Cardiac Resynchronization for Patients With Permanent Atrial Fibrillation and Narrow QRS: The APAF‐CRT Mortality Trial,” European Heart Journal 42, no. 46 (2021): 4731–4739, 10.1093/EURHEARTJ/EHAB569. 34453840

[8] J. Gabriels , D. Chang , M. Maytin , et al., “Percutaneous Management of Superior Vena Cava Syndrome in Patients With Cardiovascular Implantable Electronic Devices,” Heart Rhythm 18, no. 3 (2021): 392–398, 10.1016/J.HRTHM.2020.11.012. 33212249

[9] M. G. Bongiorni , C. Kennergren , C. Butter , et al., “The European Lead Extraction ConTRolled (ELECTRa) Study: A European Heart Rhythm Association (EHRA) Registry of Transvenous Lead Extraction Outcomes,” European Heart Journal 38, no. 40 (2017): 2995–3005, 10.1093/EURHEARTJ/EHX080. 28369414

[10] F. M. Kusumoto , M. H. Schoenfeld , B. L. Wilkoff , et al., “2017 HRS Expert Consensus Statement on Cardiovascular Implantable Electronic Device Lead Management and Extraction,” Heart Rhythm 14, no. 12 (2017): e503–e551, https://www.heartrhythmjournal.com/article/S1547‐5271(17)31080‐9/abstract. 28919379 10.1016/j.hrthm.2017.09.001

[11] D. E. Thomas , T. T. Phan , R. Hartley , N. J. Linker , D. F. Muir , and A. J. Turley , “Pacemaker Implantation in Superior Vena Cava Obstruction: Re‐Canalization, Venoplasty, and Stenting,” Journal of Cardiology Cases 14, no. 1 (2016): 29–31, 10.1016/J.JCCASE.2016.03.010. 30546655 PMC6283008

[12] N. Teo , T. Sabharwal , E. Rowland , P. Curry , and A. Adam , “Treatment of Superior Vena Cava Obstruction Secondary to Pacemaker Wires With Balloon Venoplasty and Insertion of Metallic Stents,” European Heart Journal 23, no. 18 (2002): 1465–1470, 10.1053/EUHJ.2002.3260. 12208227

[13] Y. Arora and R. G. Carrillo , “Lead‐Related Superior Vena Cava Syndrome: Management and Outcomes,” Heart Rhythm 18, no. 2 (2021): 207–214, 10.1016/J.HRTHM.2020.09.006. 32920177

[14] A. C. Liew , N. Wijesuriya , F. de Vere , S. Howell , S. Black , and C. A. Rinaldi , ““Inside‐Out” Technique to Allow Conduction System Pacing in Superior Vena Cava Obstruction,” HeartRhythm Case Reports 11, no. 2 (2025): 150–154, 10.1016/J.HRCR.2024.11.002. 40018321 PMC11862144

[15] C. S. Elayi , C. L. Allen , S. Leung , et al., “Inside‐Out Access: A New Method of Lead Placement for Patients With Central Venous Occlusions,” Heart Rhythm 8, no. 6 (2011): 851–857, 10.1016/j.hrthm.2011.01.024. 21237290

[16] R. F. Riley , S. E. Petersen , J. D. Ferguson , and Y. Bashir , “Managing Superior Vena Cava Syndrome as a Complication of Pacemaker Implantation: A Pooled Analysis of Clinical Practice,” Pacing and Clinical Electrophysiology 33, no. 4 (2010): 420–425, 10.1111/J.1540-8159.2009.02613.X. 20051021

[17] W. Mekary , E. Hebbo , A. Shah , et al., “Managing Superior Vena Cava Syndrome in Patients With Cardiac Implantable Electronic Device Leads: Strategies and Considerations,” Heart Rhythm 22, no. 2 (2025): 311–317, 10.1016/J.HRTHM.2024.06.060. 38969051

[18] M. Sohal , S. Williams , M. Akhtar , et al., “Laser Lead Extraction to Facilitate Cardiac Implantable Electronic Device Upgrade and Revision in the Presence of Central Venous Obstruction,” Europace 16, no. 1 (2014): 81–87, 10.1093/EUROPACE/EUT163. 23794614 PMC3864757

[19] C. J. Peters , W. D. Bode , D. S. Frankel , et al., “Percutaneous Balloon Venoplasty for Symptomatic Lead‐Related Venous Stenosis,” Heart Rhythm (2024), 10.1016/J.HRTHM.2024.10.010. 39393748

[20] A. Andrawos , H. Saeed , and C. Delaney , “A Systematic Review of Venoplasty Versus Stenting for the Treatment of Central Vein Obstruction in Ipsilateral Hemodialysis Access,” Journal of Vascular Surgery: Venous and Lymphatic Disorders 9, no. 5 (2021): 1302–1311, 10.1016/J.JVSV.2021.02.014. 33667742

[21] S. Gür , L. Oğuzkurt , and M. Gedikoğlu , “Central Venous Occlusion in Hemodialysis Access: Comparison Between Percutaneous Transluminal Angioplasty Alone and Nitinol or Stainless‐Steel Stent Placement,” Diagnostic and Interventional Imaging 100, no. 9 (2019): 485–492, 10.1016/J.DIII.2019.03.011. 30952526

[22] A. Schwein , Y. Georg , A. Lejay , et al., “Endovascular Treatment for Venous Diseases: Where Are the Venous Stents?,” Methodist DeBakey Cardiovascular Journal 14, no. 3 (2018): 208, 10.14797/MDCJ-14-3-208. 30410651 PMC6217567

[23] H. Mazzetti , A. Dussaut , C. Tentori , E. Dussaut , and J. O. Lazzari , “Superior Vena Cava Occlusion and/or Syndrome Related to Pacemaker Leads,” American Heart Journal 125, no. 3 (1993): 831–837, https://www.sciencedirect.com/science/article/pii/000287039390178C. 8438712 10.1016/0002-8703(93)90178-c

[24] L. Køber , J. J. Thune , J. C. Nielsen , et al., “Defibrillator Implantation in Patients With Nonischemic Systolic Heart Failure,” New England Journal of Medicine 375, no. 13 (2016): 1221–1230, 10.1056/NEJMOA1608029/SUPPL_FILE/NEJMOA1608029_DISCLOSURES.PDF. 27571011

[25] T. Kabutoya , “Superior Vena Cava Obstruction and Cardiovascular Implantable Electronic Devices—A New Era of Leadless Devices,” Mediastinum 8 (2024): 1, 10.21037/MED-23-33/COI. 38322191 PMC10839517

[26] B. S. Sidhu , J. Gould , B. Sieniewicz , B. Porter , and C. A. Rinaldi , “The Role of Transvenous Lead Extraction in the Management of Redundant or Malfunctioning Pacemaker and Defibrillator Leads Post ELECTRa,” Europace 20, no. 11 (2018): 1733–1740, 10.1093/EUROPACE/EUY018. 29452360

[27] V. S. Mehta , Y. L. Ma , N. Wijesuriya , et al., “Enhancing Transvenous Lead Extraction Risk Prediction: Integrating Imaging Biomarkers Into Machine Learning Models,” Heart Rhythm 21, no. 6 (2024): 919–928, 10.1016/J.HRTHM.2024.02.015. 38354872

[28] N. Wijesuriya , F. De Vere , V. Mehta , S. Niederer , C. A. Rinaldi , and J. M. Behar , “Leadless Pacing: Therapy, Challenges and Novelties,” Arrhythmia & Electrophysiology Review 12 (2023): e09, 10.15420/AER.2022.41. 37427300 PMC10326662

[29] J. E. Ip , M. Rashtian , D. V. Exner , et al., “Atrioventricular Synchrony Delivered by a Dual‐Chamber Leadless Pacemaker System,” Circulation 150, no. 6 (2024): 439–450, 10.1161/CIRCULATIONAHA.124.069006/SUPPL_FILE/CIRC_CIRCULATIONAHA-2024-069006D_SUPP3.DOCX. 38973458 PMC11305627

[30] P. Friedman , F. Murgatroyd , L. V. A. Boersma , et al., “Performance and Safety of the Extravascular Implantable Cardioverter‐Defibrillator Through Long‐Term Follow‐Up: Final Results from the Pivotal Study,” Circulation 151, no. 4 (2025): 322–332, 10.1161/CIRCULATIONAHA.124.071795/SUPPL_FILE/10.1161.CIRCULATIONAHA.124.071795. 39327797 PMC11771354

[31] J. P. Singh , C. A. Rinaldi , P. Sanders , et al., “Leadless Ultrasound‐Based Cardiac Resynchronization System in Heart Failure,” JAMA Cardiology (2024), 10.1001/JAMACARDIO.2024.2050. PMC11292567 39083254

[32] M. K. Elliott , P. Vergara , N. Wijesuriya , et al., “Feasibility of Leadless Left Ventricular Septal Pacing With the WiSE‐CRT System to Target the Left Bundle Branch Area: A Porcine Model and Multicenter Patient Experience,” Heart Rhythm 9, no. 10 (2022): 1974–1983, 10.1016/J.HRTHM.2022.07.017. 35940464

[33] B. S. Sidhu , J. Gould , B. Porter , et al., “Completely Leadless Cardiac Resynchronization Defibrillator System,” JACC: Clinical Electrophysiology 6, no. 5 (2020): 588–589, 10.1016/J.JACEP.2020.02.012. 32439047

